# Ventricular Neurocysticercosis: A Severe Form of the Disease Waiting for Well-Designed Therapeutic Trials

**DOI:** 10.4269/ajtmh.18-0203

**Published:** 2018-04-30

**Authors:** Oscar H. Del Brutto

**Affiliations:** School of Medicine, Universidad Espíritu Santo—Ecuador, Guayaquil, Ecuador

Neurocysticercosis (NCC)—the most common helminthic infection of the central nervous system worldwide—occurs when humans become intermediate hosts in the life cycle of *Taenia solium*, the pork tapeworm, after ingesting its eggs directly from a Taenia carrier or, less often, from the environment.^1^ Within the nervous system, parasites may be located within the brain or spinal cord parenchyma, the subarachnoid space (cranial or spinal), the sellar region, the subdural space, or the ventricular system. According to traditional pathological reports, cysticerci enter the cerebral ventricles through the choroid plexus of the lateral ventricles and then move downward within the ventricular cavities until their growth precludes further migration (most often at the level of the fourth ventricle), or when they get attached to the ependymal lining with the occurrence of a secondary inflammatory reaction, called granular ependymitis.^2,3^ Ventricular cysticerci are of different sizes and may or may not have a scolex. These cysts may be freely floating within the ventricular cavities or may be attached to the choroid plexus or the ventricular wall.

The main pathogenic mechanism explaining clinical manifestations of ventricular NCC is the obstruction of cerebrospinal (CSF) transit because of parasites or granular ependymitis occluding the foramina of Monro, the cerebral aqueduct, or the foramina of Luschka and Magendie.^4^ Patients with ventricular NCC develop a syndrome of increased intracranial pressure of subacute onset that may be associated with transient episodes of loss of consciousness or even with sudden death due to acute hydrocephalus.^5^ Focal neurological signs may also occur when parasites compress adjacent structures such as the corticospinal tracts, the periaqueductal gray matter, or the floor of the fourth ventricle. A particular clinical form of presentation due to cysts in the fourth ventricle is Bruns’ syndrome, characterized by episodic headache, neck stiffness, sudden positional vertigo, and loss of consciousness induced by rotatory movements of the head.^6^

Ventricular cysticerci are not well visualized on computed tomography, as they often appear isodense with CSF and are only recognizable because of distortion of the ventricular anatomy. By contrast, most ventricular cysts are readily visualized on magnetic resonance imaging (particularly using 3D volumetric sequences) because signal properties of the cystic fluid or the scolex differ from those of the CSF.^7^ In some cases, intrathecal administration of gadodiamide is needed to visualize hidden ventricular cysts.^8^ Spontaneous cyst mobility within the ventricular cavities or in response to movements of the head, the so-called ventricular migration sign, is reported in about 13% of patients^9^ and is currently considered a confirmatory criterion for the diagnosis of ventricular NCC.^10^

Ventricular NCC is a potentially severe form of the disease, often requiring urgent therapeutic decisions. Unfortunately, the literature on this topic is limited. Early reports suggested that cysticidal drug therapy might be effective to destroy ventricular cysts.^11,12^ However, further knowledge has, for the most part, discarded this option as complications inherent to medical treatment—mainly acute hydrocephalus due to the inflammatory reaction that follows the destruction of parasites—may be harmful to the patient. This, together with the introduction of minimally invasive surgical procedures, make surgery the preferred approach to most patients with ventricular cysts.^13,14^ In this regard, recent guidelines from a panel of experts from the Infectious Disease Society of America and the American Society of Tropical Medicine and Hygiene^15^ recommend the removal of freely floating cysts located in the lateral and third ventricles by endoscopy and removal of fourth ventricle cysts by means of microsurgical resection through a suboccipital approach. Depending on the expertise of the neurosurgeon, removal of fourth ventricle cysts can also be attempted through a supratentorial endoscopic approach, particularly when there is hydrocephalus and the cerebral aqueduct is dilated. After surgery, patients do not require subsequent cysticidal drug therapy and often do not need a shunt, unless hydrocephalus persists after surgery. If the cysts are adherent to the ventricular walls, surgical removal might be dangerous (bleeding or permanent damage of the ventricular lining may occur), and in this case, shunt placement followed by the use of cysticidal drugs is advised. Simultaneous use of corticosteroids in the perioperative period or during treatment with cysticidal drugs is also suggested. Although the strength of these recommendations is high, the quality of evidence is low because of the lack of well-designed controlled studies.

In this issue of *The American Journal of Tropical Medicine and Hygiene*, Nash et al.^16^ publish a most welcomed study on the long-term outcome of patients with ventricular NCC. The authors, working at a tertiary referral center, collected 23 patients over 33 years. Clinical presentation of the disease (most often intracranial hypertension), location of parasites (53% in the fourth ventricle), and the number of patients simultaneously presenting with other forms of NCC (including parenchymal or subarachnoid cysts) did not differ much from what is known from the literature.^9^ The main advantages of the study of Nash et al. were both detailed descriptions of cases and observations on long-term outcomes and therapeutic approaches, which—as the authors correctly observed—have changed over the years. A total of 14 patients had their cysts removed, mostly by craniotomy, and the other nine were treated by cyst fenestration or shunt placement (there is no information on the number of medically treated patients). Several patients presented with one or more complications during the perioperative period, including ventriculitis, hydrocephalus, intraventricular bleeding, and shunt failure. However, most of them recovered and at the end of the follow-up, 91% showed no evidence of active disease or recurrence of cysts; of these, 55% were asymptomatic and 30% had only mild complaints.

This is good news. The long-term prognosis of patients with ventricular NCC might not be as grim as previously suspected, as long as optimal care is provided during the acute phase of the disease and the perioperative period. This conclusion has also been suggested by another recent series of 30 patients managed with endoscopy, but that study did not report long-term patient outcomes.^14^ Further controlled studies (preferably multicentric) are urgently needed to establish the optimal surgical approach to these patients and the best rational use of corticosteroids and cysticidal drugs (in cases where surgery is not possible). In particular, studies should compare the results of open surgery versus endoscopy for treatment of freely floating fourth ventricle cysts and the optimal regimen of cysticidal drugs plus corticosteroids for patients with ventricular cysts attached to the ependymal walls.
